# Egg consumption and growth in children: a meta-analysis of interventional trials

**DOI:** 10.3389/fnut.2023.1278753

**Published:** 2024-01-05

**Authors:** Elisabeth A. Larson, Zaixin Zhao, Karlen S. Bader-Larsen, Faidon Magkos

**Affiliations:** ^1^Division of Nutritional Sciences, Cornell University, Ithaca, NY, United States; ^2^Department of Nutrition, Exercise and Sports, University of Copenhagen, Copenhagen, Denmark

**Keywords:** stunting, wasting, children, growth, egg, supplement

## Abstract

**Introduction:**

Stunting and wasting are prevalent in low- and middle-income countries, putting children at risk for disease and disability. Eggs are a nutrient-rich food that can potentially facilitate growth.

**Purpose:**

The aim of this meta-analysis was to evaluate the potential beneficial effect of egg supplementation on growth in children.

**Methods:**

Following the PRISMA guidelines, PubMed and Healthline (Ovid) were systematically searched for interventional studies on egg supplementation for growth in children aged 6 months to 18 years, with no restrictions on date. Studies were evaluated for quality using Cochrane’s GRADE technique. Data were pooled and reported as means and 95% confidence intervals.

**Results:**

Seven studies reporting on 9 unique interventions in 3,575 male and female participants were included in the meta-analysis. Participants in the intervention groups experienced significantly greater increases in height/length (by 0.47 [0.13, 0.80] cm, *p < 0.01*) and weight (by 0.07 [0.01, 0.13] kg, *p = 0.03*) when compared to those in the control groups.

**Conclusion:**

Eggs are an affordable, nutritious option for improving growth in children, though more studies with longer interventions are warranted.

**Systematic review registration**: PROSPERO (CRD42021289609: https://www.crd.york.ac.uk/prospero/).

## Introduction

In low- and middle-income countries, stunting and wasting in children remain among the most serious and prevalent consequences of poor nutrition (1). Stunting in children 0–59 months of age can be moderate or severe—defined as a height-for-age Z-score (2) that is more than 2 and 3 standard deviations (3), respectively, below the median from the World Health Organization standards—and both forms have major health consequences for those affected (1). Stunting is associated with chronic undernutrition and exposure to diarrheal disease which exacerbates nutrient losses, and can lead to neonatal death, increased susceptibility to infectious disease, and poor motor and cognitive development (4, 5). Cognitive impairment related to stunting may persist in adolescence and adulthood, limiting academic attainment and eventually impeding economic productivity and societal progress (6).

While the causes of stunting are multifaceted and may begin *in utero,* diet quality during complementary feeding and childhood is a potential contributor (7–9). Lack of dietary diversity and under-consumption of animal source foods are associated with decreased linear growth in young children, and failure to rectify undernutrition is negatively associated with catch-up growth in older children and early adolescence (9–11). Current interventions for stunting both during the critical period of early growth and the later window for potential catch-up growth rely heavily on fortified foods and nutritional supplements, and very few have evaluated potential available and affordable whole food alternatives (12). Eggs—an animal source food rich in protein, essential fatty acids, choline, vitamin A, and vitamin B12 (13)—can improve the diet quality of children (14) and potentially facilitate growth, as has been demonstrated in both observational and experimental studies (15). For example, a deficiency of choline negatively impacts lean body mass growth in infants (14, 16, 17), whereas intake of high-quality animal protein is associated with increased growth trajectory in both weight and height (18). Moreover, a large observational study in the United States found that delayed egg introduction during infancy is associated with a lower mean height-for-age Z-score and a higher risk of stunting in 6-year-old children (19).

There is an increasing interest in evaluating the effect of locally available food sources for nutrition in children. Eggs, in particular, are relatively affordable compared to other animal source foods, and homestead egg production is a feasible option for both improving the diet of the whole family and providing a secondary source of income (13). However, at present, there are few interventional trials of egg consumption and linear growth in children of any age, with inconsistent results between studies. Leveraging the increased power available through meta-analysis, we aimed to evaluate the treatment effects of pediatric egg consumption on linear growth outcomes across the currently published interventional trials.

## Methods

This review was conducted following the Preferred Reporting Items for Systematic Review and Meta-Analysis checklist (20). The protocol has been pre-registered with the PROSPERO database (registration number: CRD42021289609).

### Data sources and searches

To identify interventional trials investigating the effect of egg consumption on growth in children, we systematically searched the databases PubMed and Healthline (Ovid) on 5 November 2021. We used the following search terms with no restrictions on language, date, type of study, or place of publication: “egg” AND “children” or “child” AND “growth” or “height” or “length” or “weight.” Terms were chosen to ensure results would focus on absolute changes in height and weight, and not in shifts in the prevalence of stunting or wasting or in children’s status from stunted or wasted to non-stunted or non-wasted, respectively. Additional studies were sought by cross-referencing the bibliography of the initially identified studies and through referral by involved contributors to other relevant literature.

### Eligibility criteria

We included all interventional trials (randomized or not) in which provision of eggs alone, or eggs together with another food or supplement, was the primary intervention, and only if there was no secondary, behavioral intervention (e.g., a sanitation intervention, or dietary counseling) in conjunction with the primary, that would make it difficult to parse the sole effect of the dietary intervention. Studies were eligible if they included participants from the age of 6 months through the age of 18 years to capture children from the earliest period of complementary feeding, through the end of the late catch-up growth period of adolescence, with the intention to separate out results for young (<2 years) and older (>2 years). Studies were included if they reported outcomes of height (or length) and weight. Excluded studies were those that were observational and associational rather than experimental in nature, not written in the English language, and/or if they were secondary analyses of already included studies. Additionally, we excluded studies on egg-shell consumption, as we were primarily interested in the effect of the egg itself. We also excluded studies where increased egg consumption was a by-product of a community-based egg production intervention where egg intake was not the primary exposure and growth was not the primary outcome of interest.

### Data extraction and quality assessment

We created a data extraction tool in which the following information was retrieved from identified studies: first author and year, study design, participant information (including sample size, age, and location), methodology (including intervention duration, dose, and frequency), outcomes measured, and growth-specific results (mean ± SD). Studies were evaluated using the Grading of Recommendations Assessment, Development and Evaluation (GRADE) technique, and the overall evidence for each outcome was given a GRADE rating of either high, moderate, low, or very low. Risk of bias was assessed using the RoB-2 tool available through Cochrane’s ReviewManager (RevMan) software. We evaluated the risk of bias along the following domains: Random Sequence Generation (selection bias), Allocation Concealment (selection bias), Blinding of Participants and Personnel (performance bias), Blinding of Outcome Assessment (detection bias), Incomplete Outcome Data (attrition bias), Selective Reporting (reporting bias), and Other Biases.

### Statistical analysis

The continuous primary outcomes assessed were the change in height/length from baseline and the change in weight from baseline. Data for the analysis (means and SDs) were extracted from each article’s text, tables, and figures. When the standard error was reported in the published paper, it was converted to SD by multiplying by the square root of the sample size. When none of these data were available in the published paper, they were obtained by contacting the authors of the primary studies. We pooled data using Cochrane’s RevMan software and R statistical software for the subgroup analyses. Continuous outcomes were analyzed in RevMan and R using the mean difference with 95% confidence interval (12), and statistical significance was set at *p ≤ 0.05*.

We evaluated statistical heterogeneity using Tau-squared, I-squared, and Chi-squared measures of heterogeneity. For our purposes, an I-squared of <25% and >75% signified low level and high level of heterogeneity, respectively. An I-squared between 25% and 75% was considered to be of moderate heterogeneity. If *p > 0.05*, it was assumed that no significant heterogeneity existed. A random-effects model was used for all analyses.

Additionally, a meta-regression subgroup analysis was performed by age, excluding the two interventions conducted in children over the age of 2 years. A third analysis was performed (growth rate-adjusted analysis), normalizing the changes in growth for the duration of the intervention of each study.

## Results

### Study identification and retrieval

**Figure 1:**
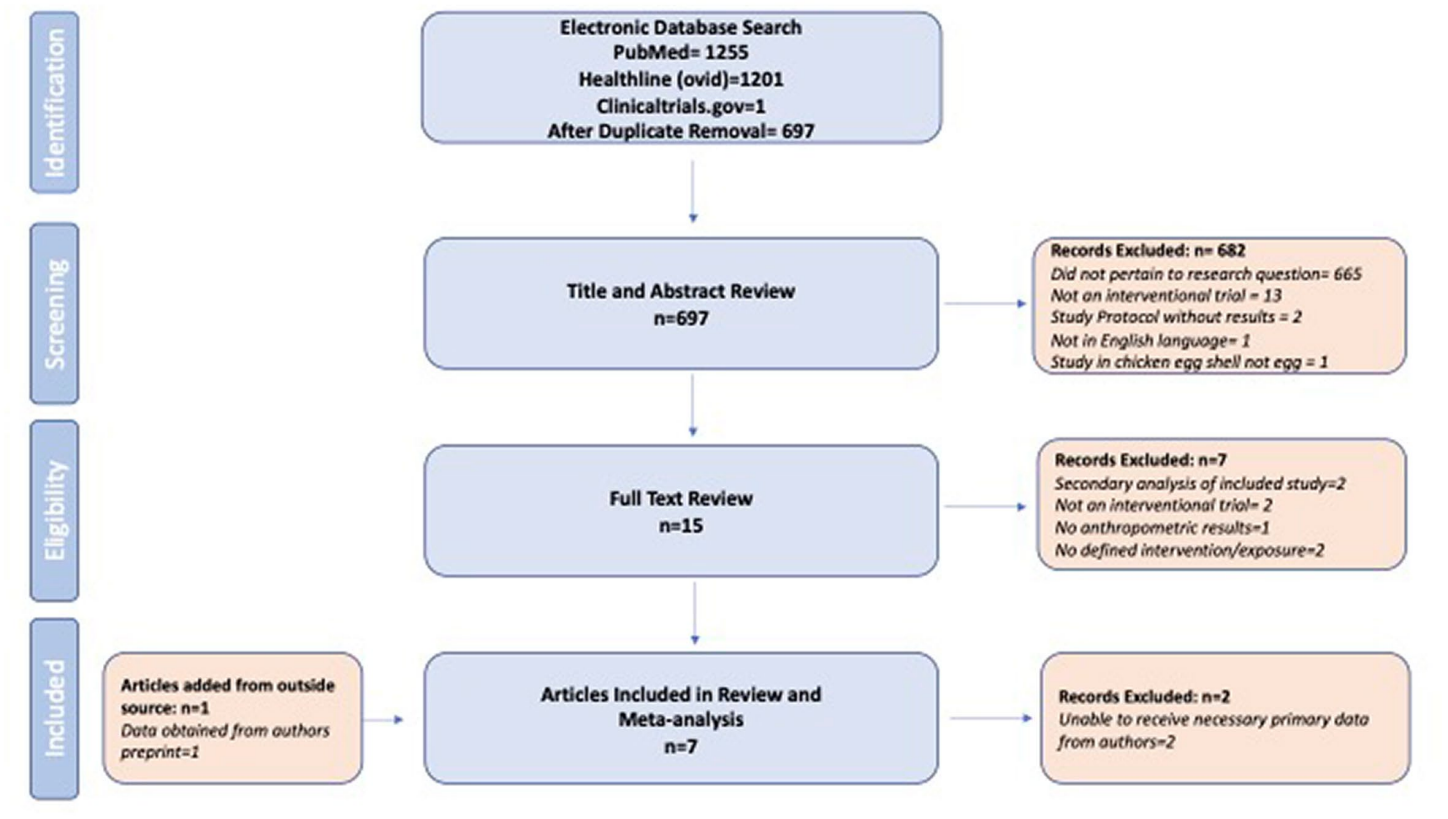
Identification and screening of included studies.

Database searches through PubMed and Healthline (Ovid) resulted in 696 unique articles after removing duplicates; the PRISMA flow diagram is shown in Figure 1. An additional article was found by searching clinicaltrials.gov, resulting in a total of 697 articles to be reviewed. The titles and abstracts of these 697 unique articles were then reviewed by one member of the research team (EAL), resulting in 15 trials for full-text review. This process was confirmed by a second member of the research team (KSBL). EAL then reviewed the full text of these 15 trials, and KSBL confirmed any decisions. Any disagreements were resolved by a third member of the research team. Later in this process, one additional study (21) was identified during an oral presentation at a scientific conference (ICN Tokyo, December 2022), and results from the final published paper were included. This resulted in 7 articles and 9 unique interventions meeting the inclusion criteria (Figure 1).

### Characteristics of included studies

Six of the 7 studies were randomized controlled trials (RCTs; Table 1). The one non-randomized intervention study used a comparator group from an observational cohort with participants who had similar characteristics (24). In total, 3,575 male and female participants were included. While Baum et al. (22) and Suta et al. (21) did not report stunting and wasting at baseline, roughly 30% of the children in the study by Bierut et al. (23) were stunted and fewer than 5% were wasted at the start of the intervention. In the study by Iannotti et al. (12), baseline stunting prevalence was 38%, and baseline underweight prevalence was 4% in the control group and 10% in the intervention group. In Mahfuz et al. (24), children were only included if they had a length-to-age Z-score < 1, and 47% in both the intervention and comparator groups had stunting (baseline rates of wasting were not reported). The baseline prevalence rates of stunting, underweight, and wasting in the study by Stewart et al. (7) were 14, 8, and 1%, respectively. In Zhao et al. (25), prevalence of stunting and wasting at baseline was not reported. The age of participants across all included studies ranged from 6 months to 14 years. Egg-alone interventions ranged from 1 to 2 eggs daily with an intervention duration of 6–8 months. Egg-plus interventions varied greatly and included vitamin A, milk, micronutrient sachets, and bovine colostrum in addition to egg. These interventions ranged in duration from 2 months to 2 years. All of the included studies were conducted in low- and middle-income countries.

**Table 1:** Characteristics of included studies.

Author	Design	Country	Participants	*n*	Age	Arms	Duration
Baum et al. (22)	RCT	Uganda	Males and females	241	6–9 years	1 egg or 2 egg supplement vs. no supplement	5 times per week for 6 months
Bierut et al. (23)	RCT	Malawi	Males and females	275	9 months	Bovine colostrum (5.7 g) and dried whole egg powder (4.3 g) vs. isoenergetic amounts of unfortified corn/soy flour (15 g)	2 times per day for 3 months
Iannotti et al. (12)	RCT	Ecuador	Males and females	163	6–9 months	1 egg supplement vs. no supplement	1 time per day for 6 months
Mahfuz et al. (24)	Non-randomized intervention	Bangladesh	Males and females	646	12–18 months	1 boiled egg, 150 mL UHT milk, and 1 sachet micronutrient powder vs. no supplement	6 days per week for 60 days
Stewart et al. (7)	RCT	Malawi	Males and females	660	6–9 months	1 egg supplement vs. no supplement	1 time per day for 6 months
Suta et al. (21)	Cluster randomized trial	Thailand	Males and Females	791	8–14 years	Whole egg v. yolk free supplement vs. no supplement	10 per week for 35 weeks
Zhao et al. (25)	RCT	China	Males and females	955	6–13 years	200 g milk and 50 g braised egg vs. usual diet	1 time per day for 2 years

RCT, randomized controlled trial; UHT, ultra-high temperature.

### Quality assessment and risk of bias

**Figure 2:**
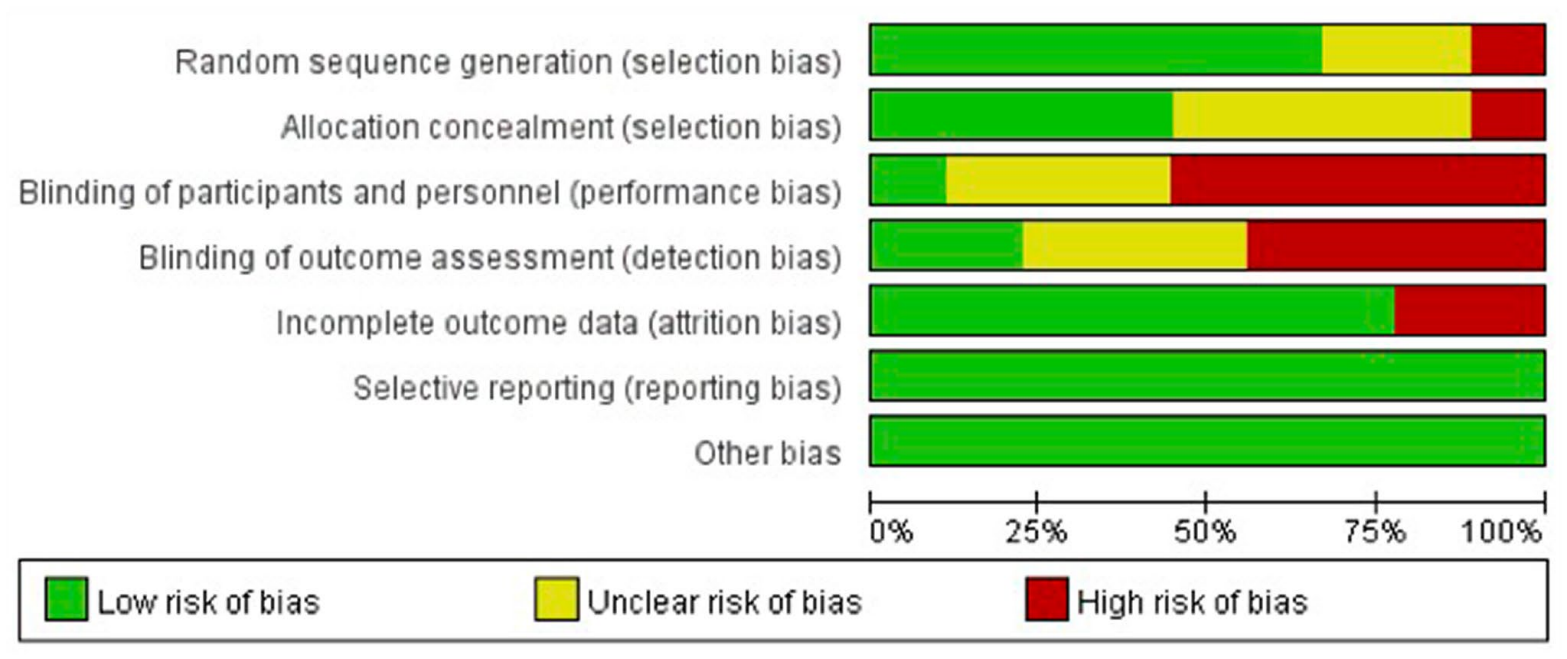
Risk of bias assessment.

The quality of the included studies as assessed by the GRADE criteria demonstrated high certainty of the evidence for the outcomes of both weight and height/length. The risk of bias of the included studies is shown in Figure 2. One study had a high risk of selection bias due to the non-randomized design (24). Four studies had high risk of performance bias as the participants were not blinded to the intervention, and three of the studies did not report whether participants and/or personnel were blinded, resulting in unclear risk of bias (7, 12, 21, 22, 24, 25). Another study had a high (25%) dropout rate overall, which also seemed to be different among the intervention arms (22). In the two-egg group, there were no dropouts; in the one-egg group, 7 participants did not complete the study (9%), and in the no-egg control group the number of participants who did not complete the study was 52 (52%) (22).

### Effect of egg consumption on change in height/length

**Figure 3:**
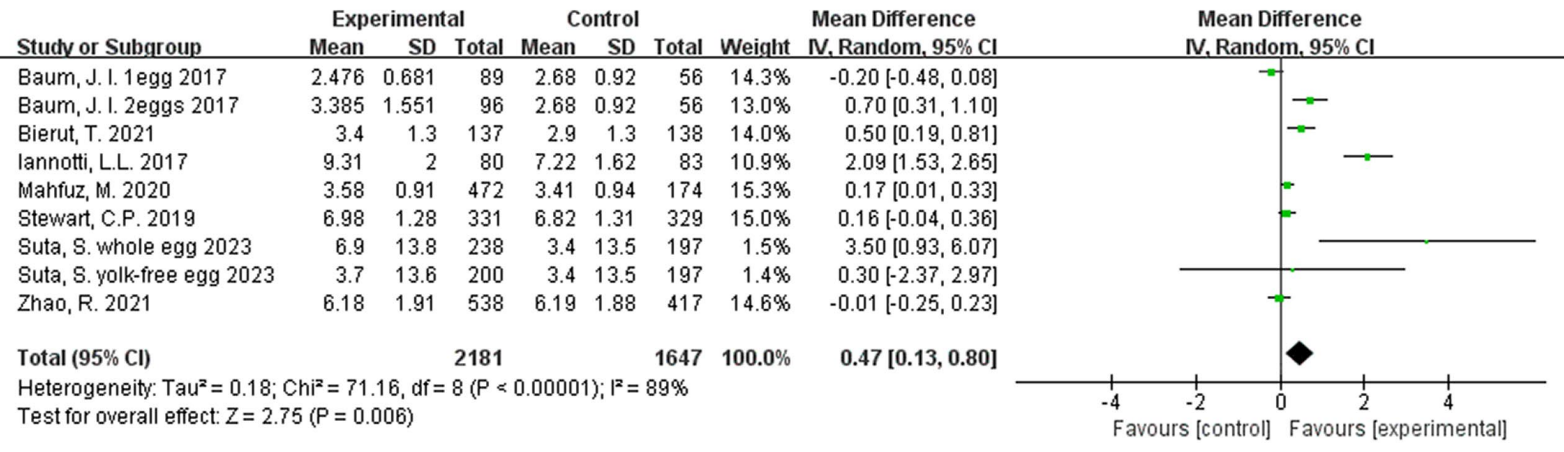
Effect of egg supplementation on height (cm).

A meta-analysis of the 9 interventions (across the 7 studies) revealed that children in the egg supplementation groups had a greater increase in height when compared to those in the control groups by 0.47 cm (95% CI: 0.13, 0.80 cm, *p < 0.01*; Figure 3). Two comparisons (22, 25) favored the control group, but these differences were not statistically significant. There was high level of heterogeneity across interventions (*p < 0.00001*; I^2^ = 89.0%; Figure 3).

**Figure 4:**
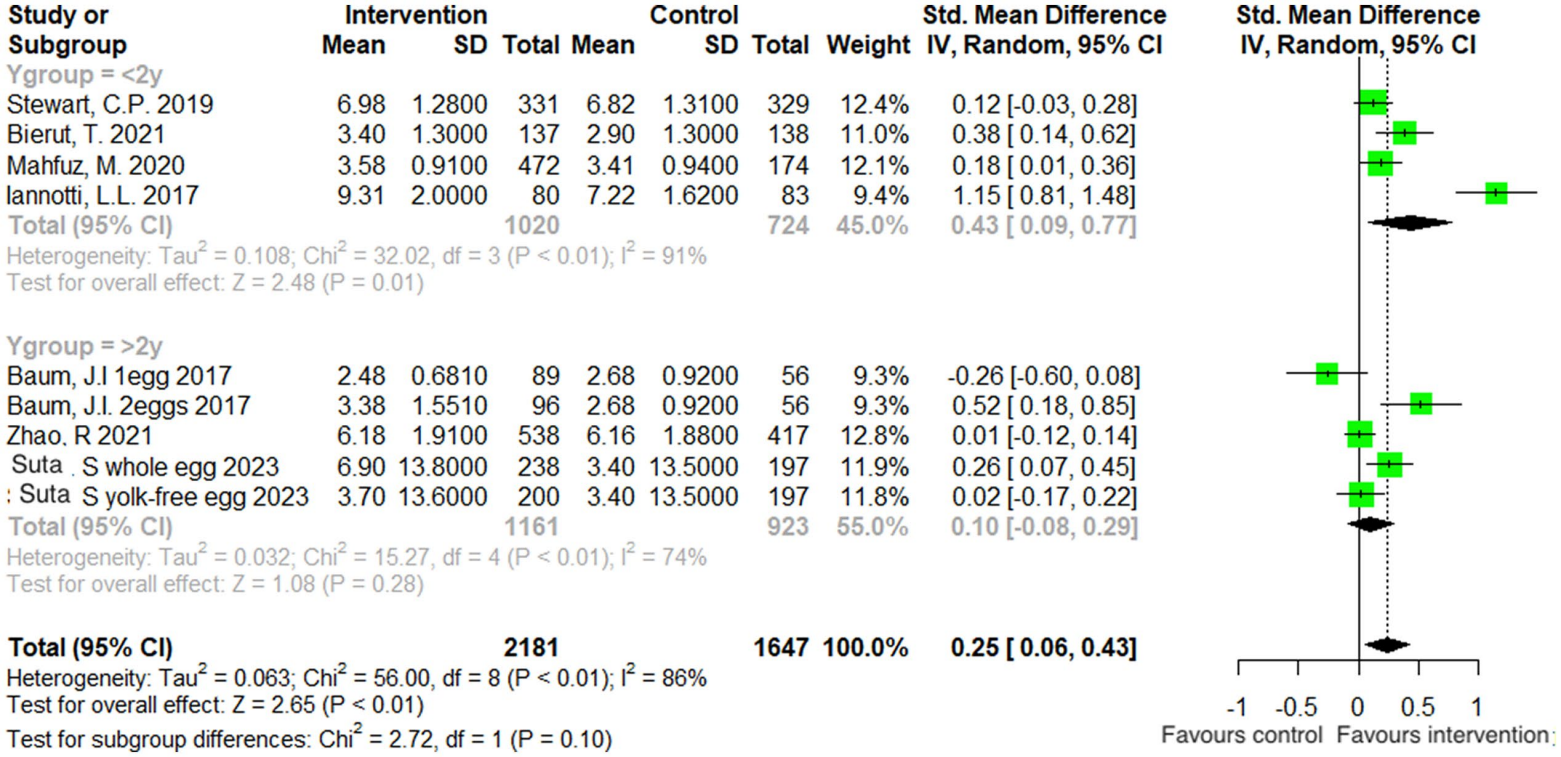
Effect of egg supplementation on height (cm)—analysis stratified by age.

A subgroup analysis was also performed, according to the age of the participants In the included studies: one with participants over the age of 2 years (4 studies), and one with participants under the age of 2 years (4 studies). When analyzed by subgroup, there was no significant treatment effect in the older age group (*p = 0.28*), but there was a significant benefit of egg supplementation in the younger age group by 0.43 cm (95% CI: 0.09, 0.77 cm, *p = 0.01*). Both subgroup analyses were characterized by substantial heterogeneity (Figure 4).

**Figure 5:**
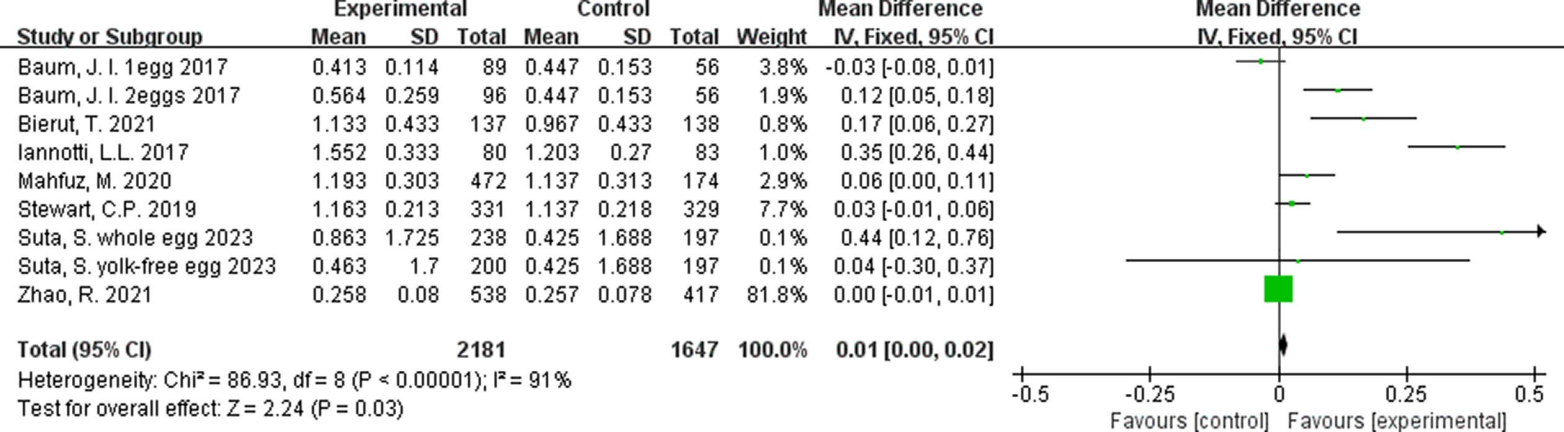
Effect of egg supplementation on height (cm/month)—analysis adjusted for intervention duration.

Additionally, a third analysis was conducted on the rate of change, to account for any differences in intervention duration across studies. Rate of change for each study was calculated as: mean difference in outcome/duration of intervention. The results of this analysis for height were marginally beneficial in favor of egg supplementation (Figure 5).

### Effect of egg consumption on change in body weight

**Figure 6:**
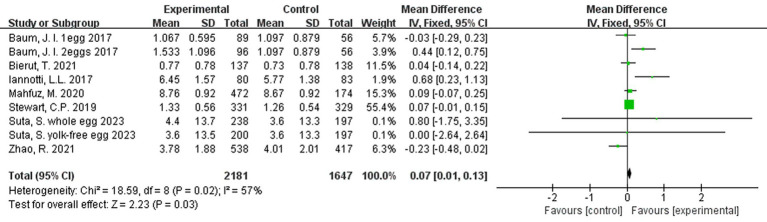
Effect of egg supplementation on weight (kg).

The meta-analysis of the 9 interventions demonstrated that children in the egg supplementation groups had a greater increase in weight when compared to those in the control groups by 0.07 kg (95% CI: 0.01, 0.13 kg, *p = 0.006*; Figure 6). Three comparisons (21, 22, 25) were in favor of the control group, but results were not statistically significant (Figure 6). There was moderate heterogeneity across interventions (*p = 0.02*; I ^2^ = 57.0%).

**Figure 7:**
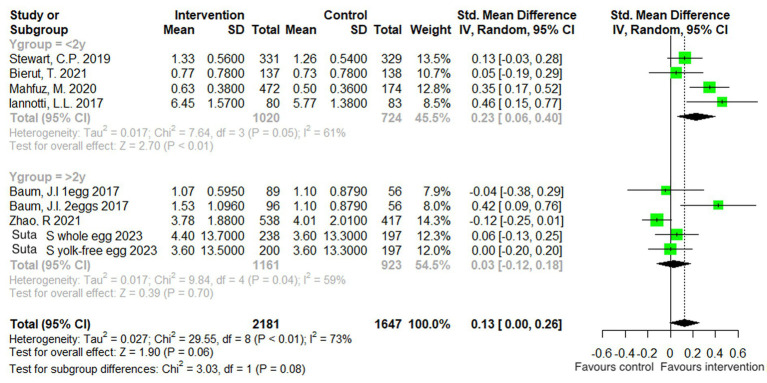
Effect of egg supplementation on weight (kg)—analysis stratified by age.

A subgroup analysis by age was also performed. Body weight was not significantly affected by egg supplementation in children older than 2 years of age (*p = 0.70*), but in the younger age group (≤2 years old), there was a significant beneficial effect by 0.23 kg (95% CI: 0.06, 0.40 kg, *p < 0.01*). There was moderate heterogeneity in both subgroup analyses (Figure 7).

**Figure 8:**
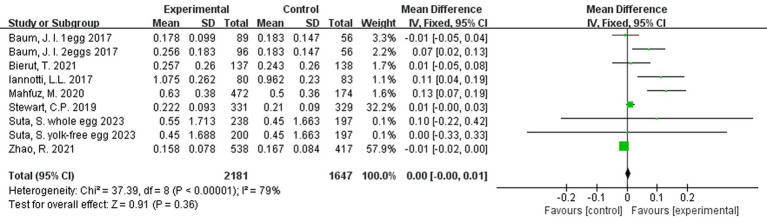
Effect of egg supplementation on weight (kg/month)—analysis adjusted for intervention duration.

In a rate-adjusted analysis for weight, there were no significant differences between groups (Figure 8).

## Discussion

This meta-analysis is the first to evaluate the effect of egg consumption, alone or as part of a multi-component supplementation, on growth in children. Overall, improved growth in terms of both weight and height was observed in the treatment groups when compared to the control groups. The overall benefit was an additional 0.43 cm in height and an additional 0.07 kg in weight. Furthermore, these beneficial changes were more pronounced in children younger than 2 years of age as opposed to those older than 2 years. These findings are consistent with the observation that earlier introduction of eggs during infancy is associated with better growth indicators in children 1–6 years old (19), and suggest that supplementation with eggs can potentially improve the nutritional status of young children in low- and middle-income countries. Similarly, they suggest an urgency for early intervention, as the results were less supportive of interventions after 2 years of age.

While the overall results from our study reveal a statistically significant beneficial effect on both children’s height and weight with egg supplementation, it is doubtful if these gains will be significant clinically, particularly given the rapid growth that occurs in children under the age of 2 years. From ages 6–12 months, the rate of growth for infants in high-income settings is about 1 cm per month, or 6 cm over the course of 6 months (26). A difference of 0.4 cm, therefore, equates to an extra 2 weeks of linear growth over a period of about 6 months, which may not be significant clinically. Similarly, weight increased by 0.07 kg in the treatment group compared to the control group, whereas the average infant is expected to grow by about 0.1 kg per week in late infancy (26). However, there is evidence that the whole population of young children (≤ 3 years old) in low- and middle-income countries experiences faltering growth relative to the international standard; therefore, increases even of a small magnitude may have clinical relevance despite not meeting the international standards (27). For these reasons, it is possible that egg supplementation would yield clinically relevant benefits in low- and middle-income settings, and particularly for children with more compromised nutritional status.

Even more so, when these effects are compared to other meta-analyses of nutrition supplementation for growth in children, their clinical relevance becomes clearer. Two other meta-analyses—one on milk and milk product supplementation (for 3 to 24 months) in children aged 6–18 years, and another one on milk protein supplementation (for 1 week to 1 year) in children aged 9 months to 12 years—found that supplementation resulted in increased weight gains of 0.48 kg (95% CI: 0.19, 0.76 kg; *p = 0.001*) and 0.42 kg (95% CI: 0.23, 0.61 kg; *p < 0.001*), respectively (28, 29). However, only the milk protein supplement produced significant results for height/length, with the treatment group benefitting from a 0.42 cm additional increase when compared to the control group (29). Importantly, the duration of supplementation (28), the age of children (28) and their nutritional status at baseline (29) were associated with the observed benefits in response to the intervention. In this sense, although the egg-associated gains found in our meta-analysis were modest, they were seen along both the axes of weight and height/length, perhaps indicating that eggs may offer better long-term nutrition and health outcomes for young children.

However, barriers remain for egg consumption by children in low- and middle-income countries. Availability and cost may play a role, as do cultural norms around food, such as which family members are prioritized to receive available food (30). While small-scale production efforts will likely not shift general population intakes, integrated education about chicken farming and nutrition has been shown to increase egg consumption at the community level, and may be a feasible approach to improving diet and growth outcomes in children (31, 32). It is also crucial that nutrition education and outreach not end at the conclusion of the study period—as evidenced by the growth faltering during follow-up after the active intervention in Ecuador (12).

There were some limitations to this work. Despite the affordability, accessibility, and efficacy of eggs as a nutrition supplement, experimental studies on their use in children in clinical trial settings are few. Furthermore, there is high heterogeneity among trials, particularly pertaining to results for height. To minimize this problem, subgroup analyses stratified by age were conducted but heterogeneity remained high. Importantly, all current research on the topic has been conducted in low- and middle-income countries. These regions often have the burden both of malnutrition and disease and infection also impacting growth, making it difficult to generalize results to higher income countries. It is conceivable that the sole presence of research conducted in low- and middle-income countries is due to publication bias, as areas with more stunting and wasting have a lower baseline from which to improve and are therefore more likely to generate significant results (29). Furthermore, it is hard to attribute the observed benefits to eggs *per se*, particularly since not all the interventions evaluated eggs alone; some evaluated eggs together with other supplements. In addition, some studies compared the active intervention against a control group that received an iso-energetic supplement (23), while most compared the active intervention against a control group that did not receive anything (7, 12, 22). It is thus possible that some of the beneficial effects of egg supplementation may have been due to the relative energy surplus. Likewise, total dietary energy intake was not reported in all studies and thus the lack of an effect of egg supplementation in some studies may have been masked by an overall dietary energy deficit and negative energy balance. Lastly, a limitation of our statistical analysis was that the control group in two studies (21, 22) was counted twice—once in comparison with the one-egg group, and once in comparison with the two-eggs group.

## Conclusion

This meta-analysis found a modest benefit to children’s growth by supplementing their diet with eggs—whether alone or together with other dietary components—for a period of 2–8 months. Whether longer periods of supplementation, or supplementation exclusively in those with stunting or wasting, will produce greater and clinically more meaningful effects remains to be examined in future studies. Larger RCTs evaluating egg consumption alone over longer periods of time are needed to precisely determine the effects of eggs on growth in children. Given that eggs are an easily accessible food in many low- and middle-income countries that can be locally produced, results of such an intervention could provide families with a realistic and manageable nutrition goal for their children.

## Data availability statement

The data analyzed in this study is subject to the following licenses/restrictions: datasets are owned by the authors of the original articles included in the meta-analysis, and may be requested from them. Requests to access these datasets should be directed to eal254@cornell.edu.

## Author contributions

EL: Conceptualization, Data curation, Investigation, Methodology, Writing – original draft, Writing – review & editing. ZZ: Data curation, Formal analysis, Software, Visualization, Writing – review & editing. KB-L: Project administration, Validation, Writing – review & editing. FM: Conceptualization, Investigation, Methodology, Project administration, Supervision, Writing – review & editing.
